# Magnetic Beads Enhance Adhesion of NIH 3T3 Fibroblasts: A Proof-of-Principle *In Vitro* Study for Implant-Mediated Long-Term Drug Delivery to the Inner Ear

**DOI:** 10.1371/journal.pone.0150057

**Published:** 2016-02-26

**Authors:** Pooyan Aliuos, Jennifer Schulze, Markus Schomaker, Günter Reuter, Stefan R. O. Stolle, Darja Werner, Tammo Ripken, Thomas Lenarz, Athanasia Warnecke

**Affiliations:** 1 Department of Otorhinolaryngology, Head and Neck Surgery, Hannover Medical School, Hannover, Germany; 2 Cluster of Excellence “Hearing4All”, Hannover, Germany; 3 Laser Zentrum Hannover e.V., Hannover, Germany; Federal University of Rio de Janeiro, BRAZIL

## Abstract

**Introduction:**

Long-term drug delivery to the inner ear may be achieved by functionalizing cochlear implant (CI) electrodes with cells providing neuroprotective factors. However, effective strategies in order to coat implant surfaces with cells need to be developed. Our vision is to make benefit of electromagnetic field attracting forces generated by CI electrodes to bind BDNF-secreting cells that are labelled with magnetic beads (MB) onto the electrode surfaces. Thus, the effect of MB-labelling on cell viability and BDNF production were investigated.

**Materials and Methods:**

Murine NIH 3T3 fibroblasts—genetically modified to produce BDNF—were labelled with MB.

**Results:**

Atomic force and bright field microscopy illustrated the internalization of MB by fibroblasts after 24 h of cultivation. Labelling cells with MB did not expose cytotoxic effects on fibroblasts and allowed adhesion on magnetic surfaces with sufficient BDNF release.

**Discussion:**

Our data demonstrate a novel approach for mediating enhanced long-term adhesion of BDNF-secreting fibroblasts on model electrode surfaces for cell-based drug delivery applications *in vitro* and *in vivo*. This therapeutic strategy, once transferred to cells suitable for clinical application, may allow the biological modifications of CI surfaces with cells releasing neurotrophic or other factors of interest.

## Introduction

For hearing sensation, the sensory hair cells act as mechano-electrical transducers and excite the primary auditory neurons, namely the spiral ganglion neurons (SGN), which in turn produce action potentials that are sent to the brain for further processing and recognition of auditory signals. In addition to the mechano-electrical signal transduction, hair cells provide a sustained source of neurotrophins to the SGN [1]. Thus, the loss of sensory hair cells, as it is the case in sensorineural hearing loss, accounts for the secondary degeneration of auditory neurons [2].

Cochlear implants (CI) are widely used to treat severe to profound sensorineural hearing loss, consisting of a maximum number of 22 active platinum electrode contacts, embedded in insulating silicone, for the direct electrical stimulation of the auditory nerve [3]. This limited number of electrode contacts takes over the task of the sensory hair cells enabling the perception of sound and open speech recognition. Despite developments in speech processor electronics and the introduction of advanced speech processing algorithms, the frequency discrimination is still accordingly poor compared to the natural hearing sensation [4–6]. Thus, speech perception in a noisy environment or in the event of multiple speakers as well as appreciation of tonal sounds such as music is difficult for patients treated with CIs. Beside the limited number of CI electrode contacts, the degeneration of target neurons (i.e. SGN) accounts for the loss of electrical stimulation selectivity and effectiveness [7]. Thence, current research targets the protection and/or replacement of SGN since greater numbers of auditory neurons are likely to improve the outcome of electrical stimulation by CIs and therefore the clinical performance of cochlear implant patients [8,9].

Numerous studies have demonstrated the efficacy of the application of neurotrophins, such as brain derived neurotrophic factor (BDNF), in the protection of SGN *in vitro* and *vivo* [10–13]. In addition, neurotrophic factors enhance the protective effects of electrical stimulation on the auditory nerve [14–17]. Since the cessation of the treatment with neurotrophins (e.g., BDNF) has been shown to lead to an accelerated decline in neural survival, their sustained application to the neural tissue should be secured [17,18]. In addition, parameters such as the amount of BDNF release as well as the area of drug elution should be well controllable to avoid non-specific nerve regeneration and neurite outgrowth [19].

In previous studies, we have already shown that NIH 3T3 cells lentivirally modified to produce BDNF proliferated on silicone surfaces of model CI electrodes and released significant amounts of BDNF *in vitro* and *in vivo* [13,20]. *In vitro*, elevated survival rates and neurite outgrowth of SGN were observed, when compared with SGN cultured without exposure to BDNF [13,20]. Moreover, after implanting the same cell-based BDNF-delivery system into cochleae of systemically deafened guinea pigs, a significant increase in SGN survival was revealed when compared to the control group [20]. Still, despite the promising approach of long-term drug delivery by means of cell-based drug delivery systems, the coating of implant surfaces such as CI electrodes reveals some obstacles. The long-term cell adhesion to such surfaces is still not reliable and standardized. Consequently, the very likely postoperative migration of drug eluting cells from the implant surfaces possesses a crucial concern to be eliminated.

The interest in magnetic particles or beads (MB) has been raised in the recent years for clinical and research purposes. As contrast agents for magnetic resonance imaging, they have already been used in clinical application [21,22]. Moreover, MB have become an indispensable tool in the field of biomedical research, e.g., for cell separation [23,24] and drug delivery [25,26]. Dynabeads are spherical microbeads consisting of superparamagnetic polystyrene possessing uniform size and defined surfaces that can be coupled with biomolecules or cells [24,27]. After being invented by Prof. Ugelstad in 1976, Dynabeads have undergone technical advancement and are nowadays used in a vast field including clinical and research applications. The use of Dynabeads to magnetically label cells and attach them onto electrode surfaces by means of electromagnetic forces might possess a feasible approach to mediate long-term cell adhesion and to avoid postoperative migration of neurotrophic- or any other factor secreting- cells. In this context, CI electrodes might be temporarily used as electromagnetic force sources (subject to further investigations). Moreover, the cell-derived amount of BDNF might be more similar to normal physiological conditions in the body compared with exogenously applied recombinant factors.

The aim of the study was to investigate the approach of immobilizing magnetically modified BDNF-releasing cells on round magnetic surfaces as models for implant electrode surfaces by using magnetic adhesion forces. We hypothesized that the viability of labelled cells as well as their BDNF production and release remain unaffected after being functionalized by MB. Therefore, murine NIH 3T3 cells lentivirally modified to produce BDNF served as a model for cell-based drug delivery and were labelled with magnetic particles via a cell specific antibody. The effects of MB functionalization on cell viability and BDNF release were investigated. Moreover, atomic force microscopy as well as bright field imaging were performed to study the impact of MB on cell morphology.

## Materials and Methods

### Labelling cells with MB-antibody complexes

Lentivirally modified murine NIH 3T3 fibroblasts, expressing either green fluorescent protein (GFP), brain-derived neurotrophic factor (BDNF) or both were used to coat small cylindrical magnets (length: 8 mm; diameter: 3 mm; purchased from supermagnete.de) serving as models for round CI electrodes. For detailed information about the lentiviral modification of the NIH 3T3 fibroblasts we refer to a previous publication [20].

Dynabeads^®^ M-450 Epoxy (Dynal, #14011, Invitrogen, Carlsbad, USA, size of the beads: 450 nm) were used as magnetic particles to label cells. The surface activation achieved by epoxy allows the formation of a complex between the beads and antibodies. The coupling of the antibody (anti-CD90.2, BD Bioscience, Franklin Lakes, USA), with the Dynabeads was performed following the manufacturer’s instructions: after washing, the Dynabeads (1 ml = approx. 4 x 10^8^ beads) were re-suspended in 0.1 M phosphate buffer at pH 7.4–8.0 and incubated with 200 μl of the anti-CD90.2 antibody (BD Bioscience) for 15 minutes in an Eppendorf tube. Thereafter, magnetic beads and antibodies were allowed to incubate for further 20 hours in the presence of 0.1% bovine serum albumin (BSA). By using a magnet, the MB-antibody complexes were collected in the Eppendorf tube and the supernatant was discarded. The antibody-coated magnetic beads were washed and re-suspended in Ca^2+^ and Mg^2+^ free phosphate buffered saline (PBS) supplemented with 0.1% BSA and 2 mM EDTA, (pH 7.4). Finally, MB-antibody-complexes were incubated with NIH 3T3 fibroblasts in order to allow binding to the surface of the cells.

### Investigating cell viability and BDNF release

For the neutral red uptake (NRU) viability test [28], unlabelled NIH 3T3 fibroblasts (controls) and cells labelled with magnetic beads were seeded at a density of 1 x 10^4^ in 96-well plates (NUNC, Langenselbold, Germany). Cells were maintained at 37°C, 5% CO_2_ in a humidified atmosphere for 7, 14 and 21 days prior to the performance of the NRU assay. Each condition was tested in three independent tests with five repetitions each (i.e., n = 15).

For the determination of the BDNF release from cells grown on cylindrically shaped magnets, three independent ELISA experiments with five samples for each condition were measured in triplets. Using a human BDNF-ELISA kit (Boster biological technology Co. Ltd, Fremont, USA) the BDNF content was measured for unlabelled cells (NIH 3T3), for cells labelled with antibody-MB-complex (NIH 3T3+Thy1+MB) as well as for cells incubated only with MB (NIH 3T3+MB) all grown on magnets as model surfaces. The BDNF-ELISA Kit was used as recommended by the manufacturer. Briefly, 100 μl of diluted standards and samples were added to a well of the pre-coated 96-well plate. After 90 min of incubation the solutions were discarded. Then, the diluted biotinylated anti-BDNF antibody was directly added without washing and the plate was incubated for 60 min. After three washing steps with 0.01 M PBS (8.5 g NaCl, 1.4 g Na_2_HPO_4_, 0.2 g NaH_2_PO_4_ ad. 1 l dest. H2O; pH = 7.2–7.6), the provided ABC solution was added to the wells followed by an incubation time of 30 min. The plate was then washed for 5 times and the provided colour developing solution (3,3’,5,5’-tetramethylbenzidine, TMB) was added. The colour change was stopped with the provided TMB stop solution after 15–30 min. All mentioned incubation steps were performed at 37°C. Finally, the absorbance was measured at 450 nm using a photometric plate reader (Multiskan Ascent plate reader, Thermo Scientific Inc. Waltham, USA). The sample dilution buffer served as blank and all measured data were blank corrected for analysis.

### Atomic force microscopy (AFM)

A Nanowizard II AFM from JPK-Instruments AG (Berlin, Germany) was used to characterize cell morphology after incubation with MB. The cells functionalized with MB were incubated in Petri dishes (tissue culture dish 40, TPP, Switzerland) for 24 h and 48 h and were thereafter fixed using 4% PFA (diluted in cell culturing medium 1:1) for 10 minutes. Afterwards, cells were rinsed several times with PBS, were placed onto a Petri dish heater (PDH, JPK-Instruments AG) and were mounted on top of an inverted microscope (AxioObserver A1, Zeiss, Jena, Germany) for optical control. A clean Biotool XXL cantilever (Nanotools GmbH, Munich, Germany) with a nominal force constant of 0.32 N/m and tip length of ca. 10 μm was installed onto the AFM and was inserted into the PBS-filled Petri dish using the AFM stepper motors. The cantilever was allowed to rest in the buffer for at least 45 min before it was calibrated to measure its sensitivity and finally force constant using the thermal noise method [29]. The measured force constant values of cantilevers used in this study varied in a range between 0.18 and 0.56 N/m. After the cantilever was calibrated, a cell was selected using the inverted microscope and was imaged using the AFM. Experiments to investigate cell surfaces’ topographies were done in PBS and in contact mode using setpoints of ca. 1 nN, line rates of 0.15–0.25 Hz for scan fields of 100x100 μm^2^ with a pixel value of 512x512. In order to ablate cell membranes at certain areas selectively with the cantilever tip, the setpoint was elevated to ca. 5 nN during the scanning of cell surfaces for observing the cytoplasmic position of MB in fibroblasts. All images were processed using the JPK SPM data processing software (v. 4.3.25).

### Bright field imaging

In addition to the AFM, an Axiovert 200 (Carl Zeiss AG, Jena, Germany) equipped with a CCD-camera was used to verify the location / interaction of MB and the cells. Bright field images were recorded through a 100x oil immersion objective (Carl Zeiss AG, “Plan-Neofluar”, NA = 1.3).

### Statistics

Statistical analysis was performed using ANOVA and Tukey’s post-test. P-values less than 0.5 were considered statistical significant. All values are depicted as mean ± SEM.

## Results

**Fig 1:**
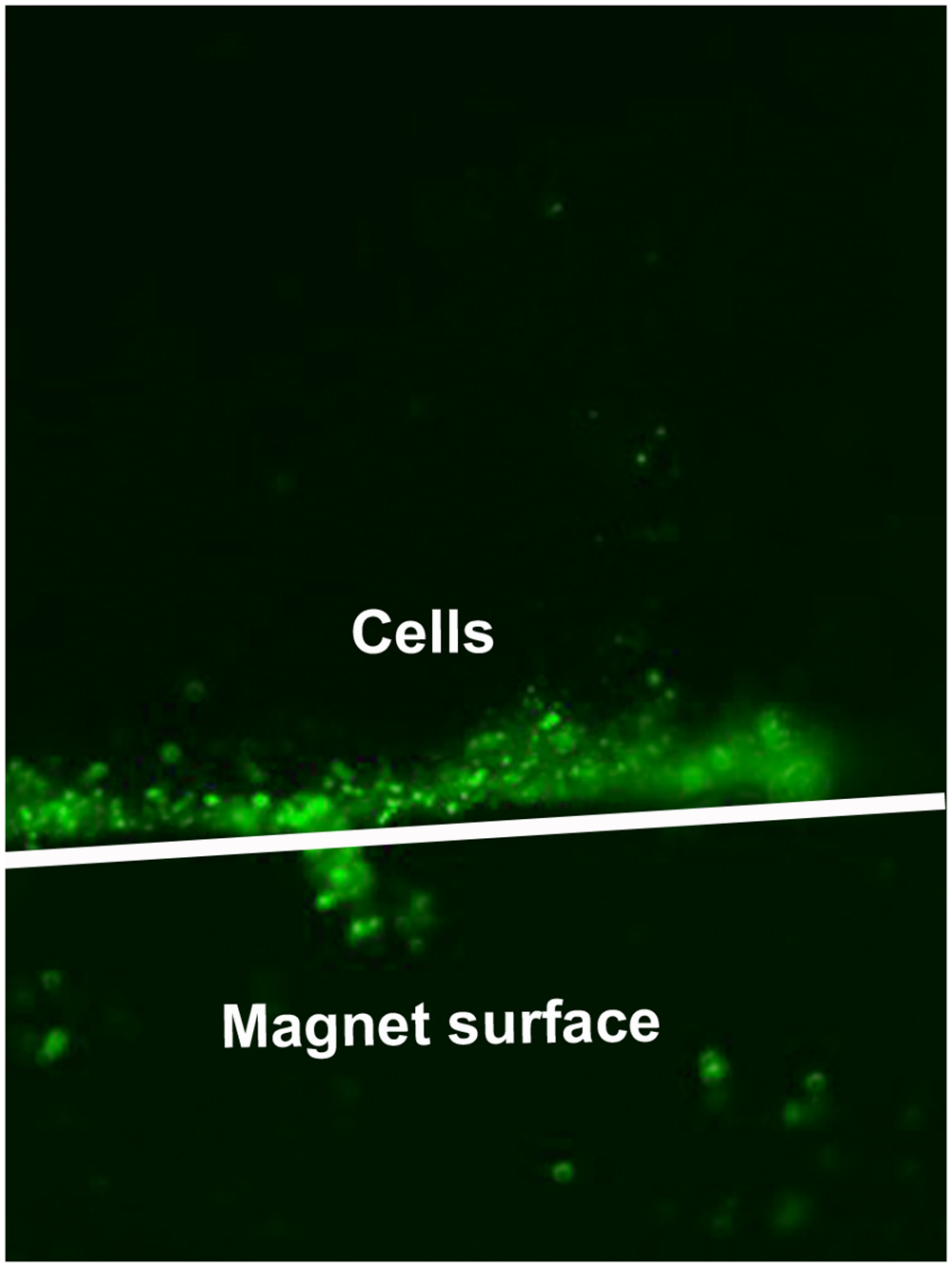
Cells labelled with MB grown on magnets. Immunomagnetically labelled NIH 3T3 fibroblasts (NIH 3T3+Thy1+MB) grown on cylindrically shaped magnets (borders outline in white; 6 mmx3 mm, LxD) cultivated in a Petri dish. Micrograph was taken 20 h after seeding. Magnification: 40x.

Murine NIH 3T3 cells lentivirally modified to produce BDNF were labelled with magnetic beads via a cell specific antibody. Labelled cells adhered onto magnetic surfaces and proliferated over the investigated period of 21 days (Fig 1).

As shown in Fig 1, most of the fibroblasts that were labelled with MB remained at the magnet surface after 20 h of cultivation, which indirectly confirms the successful labelling of the cells and their attraction by the magnetic field.

Using the neutral red uptake assay, cell viability of labelled and unlabelled cells was assessed. In addition, the influence of the labelling with MB on the production and release of BDNF from cells was proven by quantification of BDNF in cell supernatants. The localization of the beads on the cell surface as well as in the cytoplasm was investigated with AFM.

### Cell viability

The effects of MB on cell viability were examined by utilizing the neutral red uptake (NRU) assay. Therefore, neutral red dye was added to unlabelled and MB-labelled cell cultures incubated for seven, 14 and 21 days. These cultures consisted of NIH 3T3 cells unlabelled or labelled with the anti-Thy1-MB complex. Three hours after the addition of the neutral red dye, the uptake was quantified at 540 nm by means of a Multiskan Ascent plate reader (Thermo Scientific Inc. Waltham, USA). The data were normalized to the extinction (mean value) of the unlabelled (control) cells.

**Fig 2:**
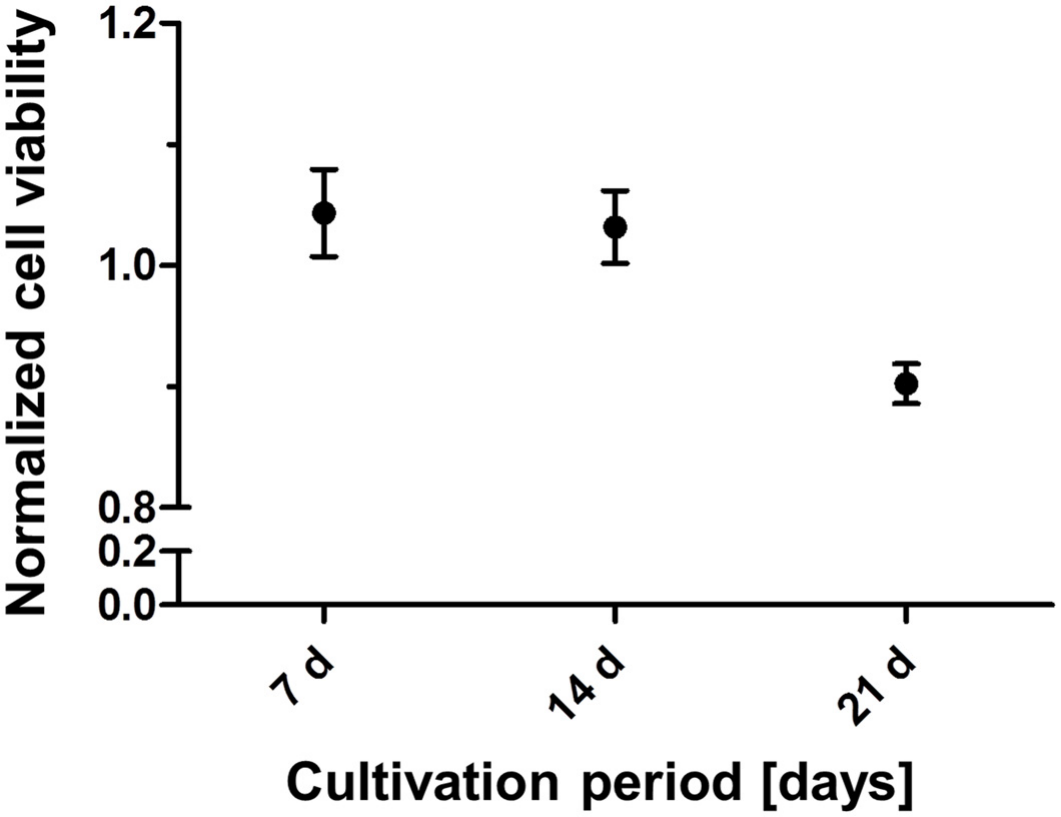
Cell viability with NRU. Normalized cell viability of NIH 3T3 fibroblasts labelled with Dynabeads in relation to the control (unlabelled cells) determined via the neutral red uptake (NRU) test. Cell viability was approved by the experiments at all different time points (7, 14 and 21 days) of cultivation after labelling with Dynabeads and remained over 85%, indicating nontoxic properties of Dynabeads.

The cultivation of fibroblasts with MB did not influence the NRU after 7 and 14 days. After 21 days of cultivation, a slight but not significant decrease was revealed in NRU, and respectively in cell viability (Fig 2). Nevertheless, since the relation between NRU of labelled to unlabelled cells (ca. 85%) did not fall below a threshold of 70% -defined by the European standard for cytotoxicity test (ISO DIN EN ISO 10993–5) as the lowest limit for cell viability- the non-cytotoxic properties of Dynabeads on NIH 3T3 fibroblasts were proven.

### BDNF production

After fibroblasts were cultured for 48 h, the amount of secreted BDNF into the medium by cells was quantified using ELISA method. Therefore, cells (unlabelled, labelled with the antibody-MB-complex (NIH 3T3+Thy1+MB) or only with MB (NIH 3T3+MB)) were seeded on round cylindrically shaped magnets and the amount of BDNF was evaluated quantitatively after 48 h of cultivation by ELISA.

**Fig 3:**
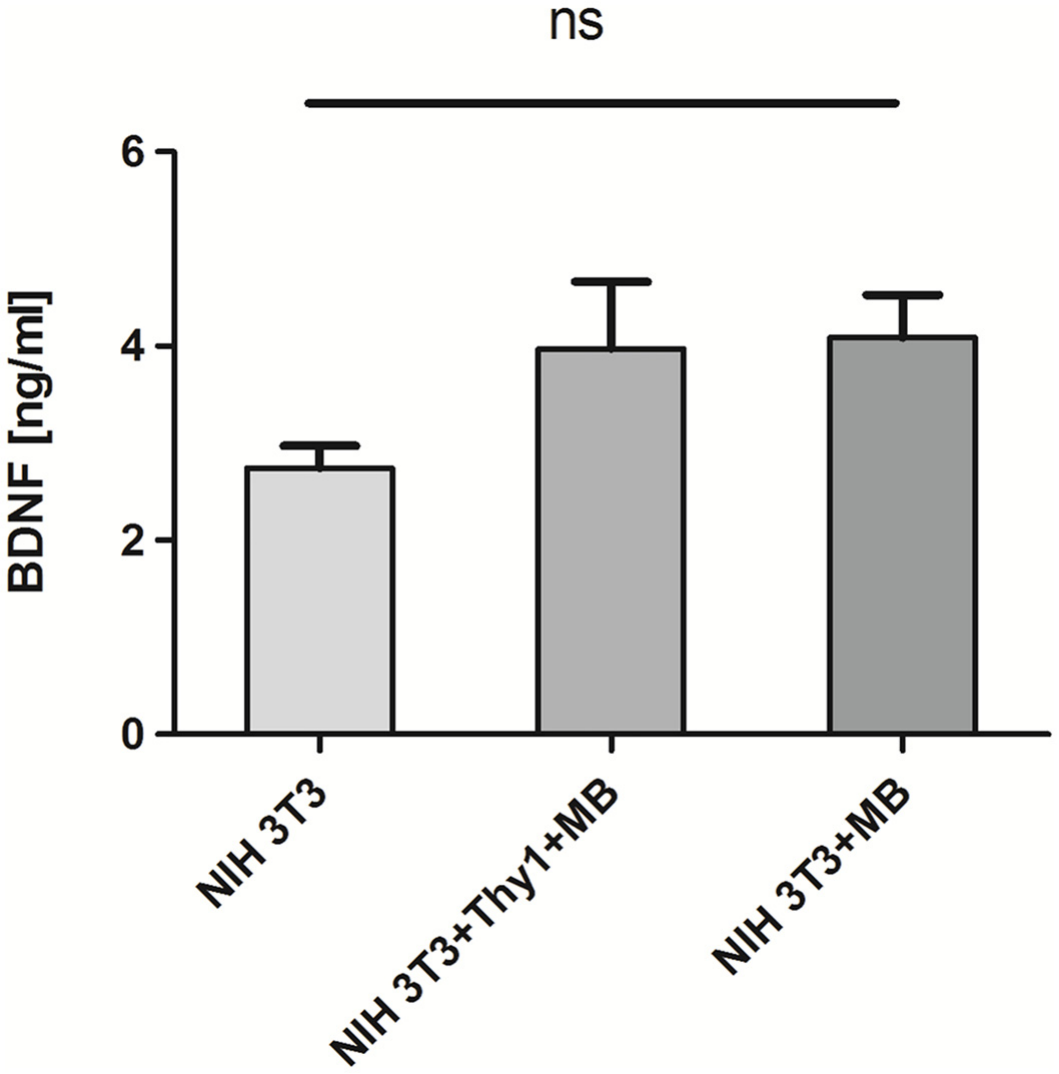
BDNF release. Release of BDNF from genetically modified NIH 3T3 fibroblasts. Unlabeled (NIH 3T3) cells were compared with cells incubated in the presence of Dynabeads-antibody-complex (NIH 3T3+Thy1+MB) and Dynabeads without antibody (NIH 3T3+MB). The BDNF release was slightly (statistically not significant) increased for cells modified with Dynabeads with or without antibody.

Fig 3 illustrates the amounts of released BDNF by native and modified fibroblasts into the cell culture medium after 48 h of cultivation. While unlabelled cells released a BDNF amount of 2.74 ± 0.23 ng/ml (mean ± SD) into the medium after 48 h of cultivation, cells labelled with Dynabeads both with and without Thy1-antibody showed slightly but not significantly elevated BDNF secretion (3.97 ± 1.54 ng/ml and 4.1 ± 0.98 ng/ml, respectively).

### Atomic force microscopy

**Fig 4:**
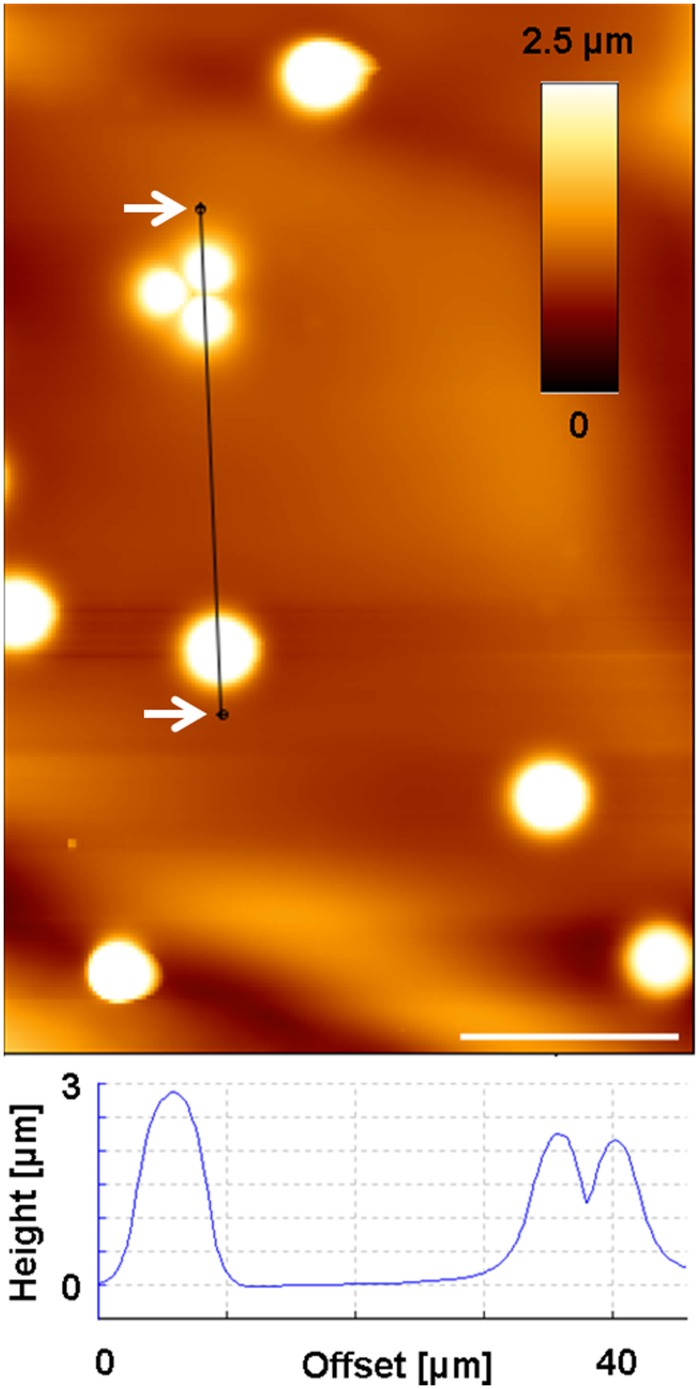
Height profile of MB. AFM height image of Dynabeads confirmed the variation of beads sizes around their nominal size of approx. 4.5 μm. Dynabeads were glued to a glass surface and were scanned using the AFM to determine their size. Scale bar: 10 μm.

AFM was employed to examine the impact of MB on cell morphology, their attachment on cell surfaces and to observe their location in the cytoplasmic environment of NIH 3T3 fibroblasts. Therefore, first the characterization of MB with the AFM was aimed, in order to be able to differentiate them from other obstacles within or on the surface of cells. Thus, the MB solution was given on microscopy glass slides and was allowed to dry in air at room temperature before AFM was used (same parameters as for investigating cell surfaces) for imaging the MB. Fig 4 illustrates the height profile of such MB examined by the AFM, revealing wide range of the dimension of MB (between ca. 2.5–6 nm) around the given nominal size by the manufacturer (4.5 μm).

**Fig 5:**
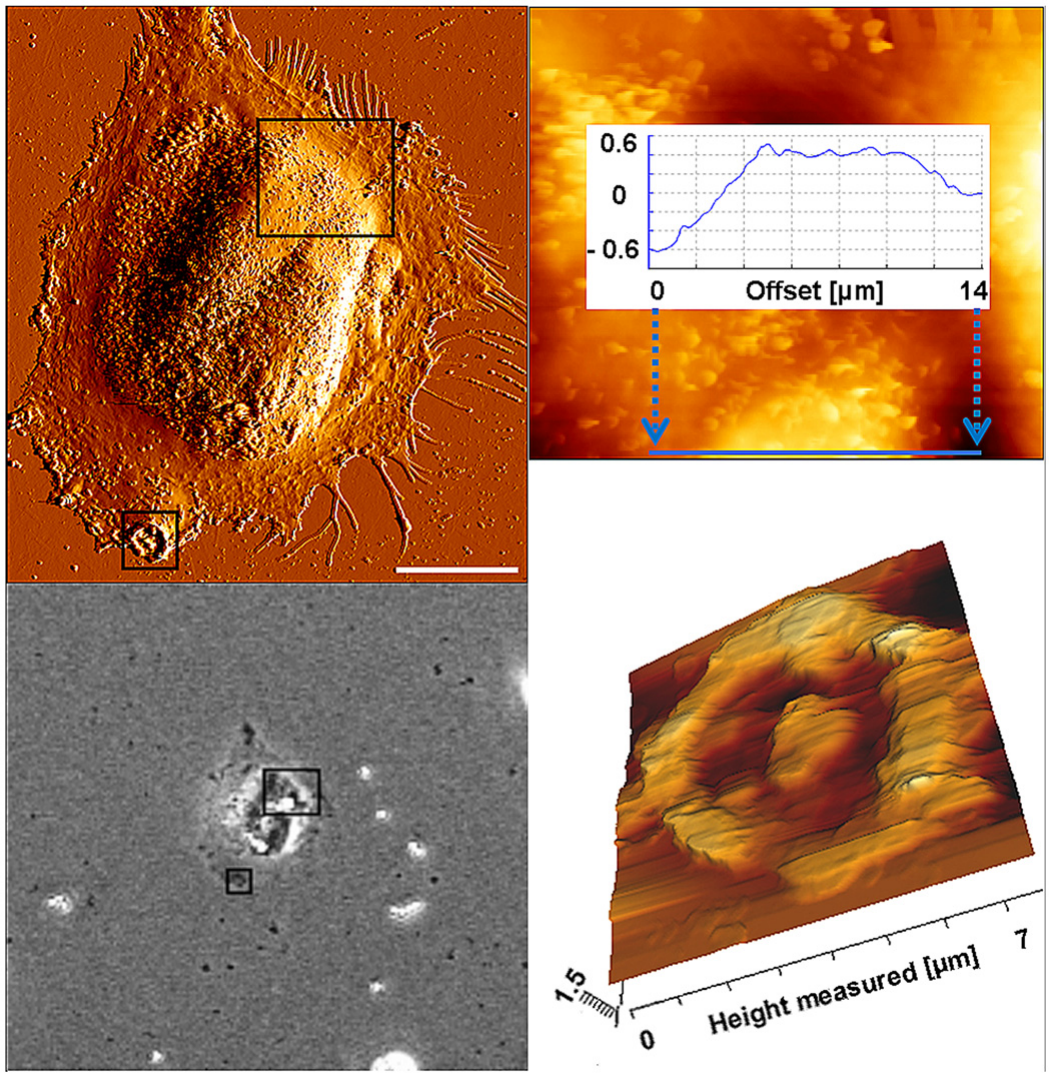
Location of MB after incubation with cells for 24 h. AFM error signal (A) and a phase contrast image (B) of a single NIH 3T3 fibroblast after incubation with magnetic beads for 24 h. C) Section of the larger rectangle of 3 A and B in an AFM height image and a cross section (blue line) over one of the magnetic beads. Correlation between the images B and C show how magnetic beads were located within the cell around the nucleus. (D) AFM height image of the section of the smaller rectangle in Figs 5A and C shows the anchor region of a single magnetic bead at the cell periphery that was removed by the cantilever from the surface of the cell. Scale bar in A: 20 μm.

**Fig 6:**
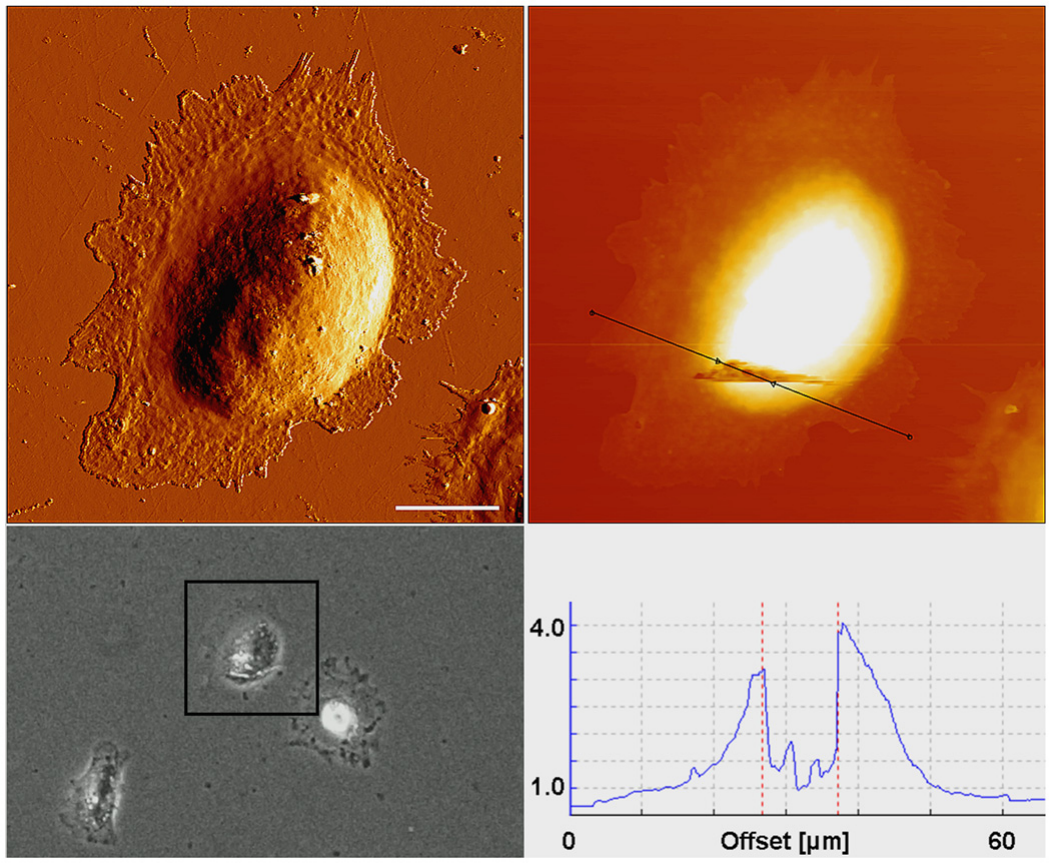
Location of MB after incubation with cells for 48 h. AFM error signal (A) and phase contrast image (B) of a single fibroblast after 48 h incubation with magnetic beads, revealing several MB at its surface (white arrows) and other MB positioned within the cell. Fig 6C shows the AFM height image of the same cell that was imaged with a higher setpoint of 5 nN to ablate the cell surface in order to observe the intracellular positioning of the beads. A cross section (black line in Fig 6C) over the degraded cell surface was used to quantify the dimensions of the observed beads (D). The two broken dashed lines in the cross section (D) indicate the borders of the anchor region of two magnetic beads that were located within the cytoplasm prior to the degradation of the cell surface by the cantilever and were disposed by it afterwards.

After having examined the characteristics of the MB, light and atomic force microscopic investigations were carried out to characterize cell morphology and position of MB in cells. Regular cell morphology was revealed after incubation with antibody-MB complex for 24 and 48 h (Figs 5 and 6).

Magnetic beads were visible under both microscopic conditions (Figs 5A, 5B and 6A, 6B). While most of the beads were located within the cytoplasm around the nuclei (Figs 5C and 6C), other beads remained at the surface of the cells (Fig 5D). These weakly bound MB were removed by the cantilever after two to three scanning procedures (Fig 5D).

### Bright field microscopy

**Fig 7:**
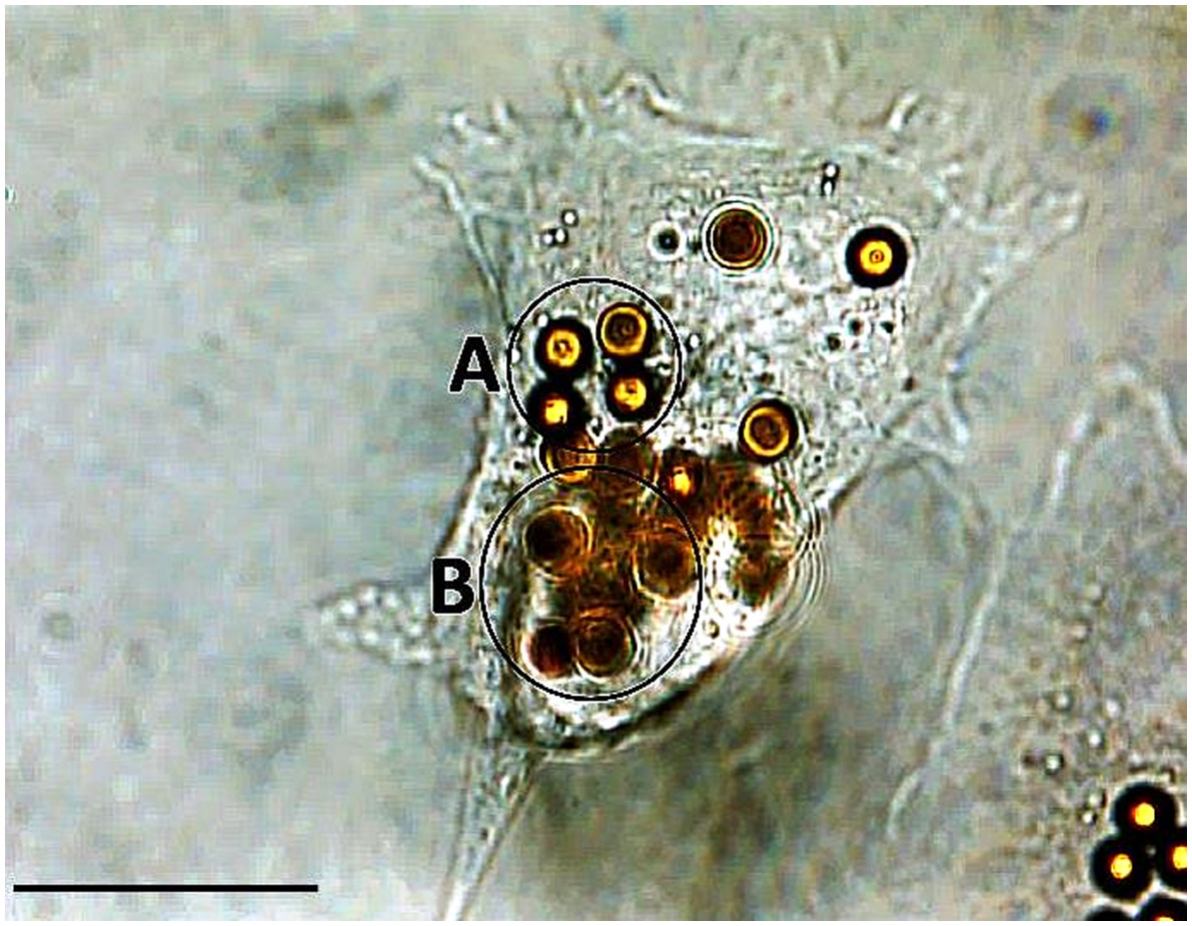
Bright field imaging. Bright field microscopic image showing a single fibroblast in the middle of the image as the host for several MB. The focus of the image reveals the cell body including its periphery and some of the beads that can be seen in focus (e.g., circle A) than the remaining MB. Other beads are not in the focal plane (e.g., circle B) indicating two different planes of the vertical positions of MB. Scale bar indicates 20 μm.

Bright field microscopy was performed to confirm the AFM results regarding the position of MB within the cytoplasm or at the surface of fibroblasts. Investigations revealed the ability of single NIH 3T3 fibroblasts to incorporate several MB (Fig 7, circle A) or to be attached by several MB at their surface (Fig 7, circle B). This was concluded since all the MB were not found to be within the optical plain / focus of the image that shows cell bodies and their periphery as well as some of the MB more focused than the environment. In other words, some MB were located at the same vertical plane as the cell body (circle A in Fig 7), while other ones were positioned at another vertical plane (circle B, in Fig 7) most probably at cell surfaces and were therefore not as focused as the first ones.

## Discussion

In the present study, a novel approach to functionalize magnetic surfaces, as a model for any implant surface capable of exposing magnetic fields, e.g., cochlear implants, with fibroblasts lentivirally modified to locally release BDNF to the surrounding neural tissue was tested.

Sustained systemic delivery of neurotrophic factors to the inner ear for maintaining auditory nerve fibres and SGN is thought to be problematic because of their short serum half-life and due to their inability to permeate the blood cochlea barrier [30]. One option to overcome this obstacle may be the use of the CI as drug delivery device within the scala tympani of the inner ear [31–33]. Nevertheless, the elution and the amount of the secreted drugs/growth factors need to be well controllable and kept within the physiologically relevant range. This is important in order to avoid unspecific cell/tissue stimulation by exaggerated secretion of drug/growth factor leading to harmful side effects [19]. In this context, cell-based drug delivery seems to be a promising approach for the sustained local application of bioactive substances [34]. Nevertheless, strategies for the secure binding of cells onto implant surfaces avoiding postoperative cell migration have still not been introduced. Therefore, the aim of this study was to investigate, if the application of magnetic fields to magnetically-labelled cells can overcome this issue by keeping them in place using magnetic forces. Dynabeads were used to label BDNF-secreting NIH 3T3 fibroblasts as a cell model. Small cylindrical magnets served as magnetic model surfaces to attract magnetically labelled fibroblasts. The objective of the study was to evaluate the extent of the cytotoxicity of the Dynabeads, their effects on cell morphology and BDNF-release as well as their ability to attach to the cells’ surfaces. To our knowledge, this is the first report on binding cells labelled with superparamagnetic beads to model surfaces possessing magnetic fields for an envisaged localized, cell-based drug delivery to confined organs mediated by the implant’s surface for application in CIs.

The NRU tests to determine the viability of cells after being cultivated at different time points in the presence of MB revealed no cytotoxic effects of MB on NIH 3T3 fibroblasts. As shown in Fig 2, the level of cell viability on the whole time axis (> 85%, Fig 2) remained above 70%, defined by the European standard (ISO DIN EN ISO 10993–5) as the lowest threshold for cytocompatibility. Since the system is envisaged to be developed for long-time application of growth factors, the presence of MB at cell surfaces or within the cytoplasm over long periods of time is crucial for keeping the cells on electrode surfaces. Therefore, bright light and atomic force microscopy were employed to investigate the position of MB and the developments in their movement within the cytosol or cell surfaces. As can be observed in Fig 7, several MB could be revealed in the proximity of single fibroblasts. Since MB were found to be at different vertical planes in the image, it was concluded that some MB were incorporated by the fibroblasts. Thus, AFM was carried out to support the bright field microscopy to investigate, if several Dynabeads with a nominal size of ca. 4.5 μm could be internalized by single fibroblasts. AFM results confirmed bright light microscopy and showed that beside some MB present at cell surfaces, most of them were internalized by the fibroblasts after 24 h and 48 h (Figs 5 and 6, respectively). In order to exclude the possibility that MB were localized under the cells, first a single cell was imaged (Fig 6A), and then the AFM experiment was modified (please see materials and methods section for more info) for ablating the cell surface at certain regions of interest to have a look inside the same cell (Fig 6C). Fig 5 shows an exemplary cell that possessed several MB at its surface (white arrows in the image). Furthermore, by comparing the light microscopic image (Fig 6B) and the AFM image (Fig 6A) it was concluded that other MB, which were not at the surface of the cell, could only be positioned either within the cytosol or under the cell. After ablating the cell surface above those MB, the AFM image of the intracellular parts of the cell showed two round indentations within the cytosol with the same diameter as given for the MB by the manufacturer (ca. 4.5 μm) (Fig 6C and 6D), delivering the evidence of cytosolic positioning of some MB. The internalization of microbeads has already been reported in several studies. For example, Dynabeads 280 with nominal diameters of 2.8 μm were shown to be phagocytosed by a murine macrophage monocyte cell line within a few hours *in vitro* [35]. Burkhardt and Merker (2002) also revealed the incorporation of beads (diameter 1 μm) -immunolabelled with the antigen CD8- by phagocytosis during a cell sorting procedure only after 45 minutes of incubation with lymphocytes [36]. To our knowledge, this study reveals the internalization of Dynabeads with relatively large size of 4.5 μm by fibroblasts for the first time. Since fibroblasts do not belong to the group of phagocytes such as neutrophils and macrophages, the within this study occurred internalization of beads is considered to be due to the endocytosis. Nevertheless, this unexpected endocytosis of the Dynabeads allows stronger accessibility of labelled cells for external magnetic field over long-term periods and counts as a further benefit of our task.

Neurotrophic factors are key players in the maintenance and survival of neuronal tissue. In the mature mammalian inner ear, the two neurotrophins BDNF and NT3 have been shown to support the survival and maintenance of the auditory neurons [37]. One of the main reasons for the degeneration of the peripheral auditory fibres and SGN is the decline of sensory hair cells and thus the loss of neurotrophic support from these neurotrophin secreting cells [38]. The performance of CI depends strongly from the remaining nerve fibres and SGN that can be electrically stimulated. Therefore, the application of neurotrophins in combination with CI may increase the quality of hearing perception in profoundly impaired patients. The use of CI electrodes as drug delivery system has been reported to lead to an enhancement of SGN survival [17,33]. Nevertheless, in those systems the electrode surfaces were coated with neurotrophic factor depots (i.e., hydrogels) and thus, showed limitations in the amount of growth factors that can be loaded as well as in the duration of growth factor application.

The amount of released BDNF of unlabelled and MB-labelled fibroblasts in the present study varied in a range between ca. 2.5 and 4 ng/ml (Fig 3). Similar amounts were released from the same BDNF-releasing cell line grown on silicone elastomers in a previous study and this amount was sufficient to protect SGN [20]. Interestingly, labelled cells showed higher amounts of released BDNF (ca. 4 ng/ml) when compared with unlabelled fibroblasts (ca. 2 ng/ml) (Fig 3). Since magnetically labelled fibroblasts may have adhered stronger onto the cylindrical magnets in response to the magnetic field, the slight increase in the secreted BDNF amount is thought to be a result of higher cell numbers and consequently higher amounts of BDNF in the media when cells were labelled with MB compared with unlabelled cells. The labelling with MB did not affect the BDNF release from the cells (Fig 3). The clinically relevant dosage of recombinant BDNF has been reported to be 50 ng/ml for the protection of SGN in the inner ear [39] and also for retinal ganglion cells of the eye [40]. Though, cannula or tube based delivery systems needed significantly higher concentrations of recombinant BDNF ranging from 50 ng/ml [12] to 100 ng/ml [11,17,41] to maintain SGN and initiate the regeneration of their neurites. These values are much higher than those measured within this study (2.5–4 ng/ml). Nevertheless, the highest measured BDNF concentration was 9.09 ± 1.97 ng/ml after 14 days of cultivation [13,20] in our previous study using the same BDNF-releasing cell line as coating for cylindrical silicone elastomer model surfaces. These studies have demonstrated that cells attach to and survive on implant surfaces for up to three weeks and are able to release BDNF over this time period. Still, less than this concentration was sufficient to induce neurite outgrowth in SGN and to enhance SGN survival *in vitro* and *in vivo* [20]. These discrepancies in biological effects between BDNF secreted from cells and human recombinant purified BDNF on SGN may be the result of differences in protein structures of both neurotrophic factors interacting with their corresponding receptors tyrosine kinase (Trk) B and p75NTR [20]. Thus, slow but sustained release of BDNF from the cells magnetically attached to the surface of the electrode may be more beneficial to SGN rather than a single shot administration of BDNF. However, the long-term release of BDNF from cells magnetically attached to implant surfaces and its bioactivity needs to be determined in future studies.

Using other systems based on hydrogel depots as implant coatings for recombinant BDNF delivery, the release showed a descending exponential curve with the maximum in the very first days and vast decrease in the secretion afterwards reaching a low plateau remaining for some weeks until the depot was exhausted [42,43]. Thus, because of the accelerated decline in neuronal survival after cessation of BDNF treatment [17,18], long-term and consistent application of growth factors should be envisaged.

## Conclusions

The cell-based drug delivery system presented here introduces a novel approach to overcome this inconsistency in the secretion of growth factors and can be used as a reliable system for delivering drugs/growth factors over long periods of time at a clinically relevant concentration. Furthermore, the amount of released growth factors can be modified by varying the number of growth factor eluting cells attached to the implant surface. Further studies should concentrate on the transient generation of electromagnetic fields using CI electrodes. During the healing period prior to activation of the CI, a sustained delivery of BDNF would provide neuroprotective and neuritogenesis inductive stimuli to the SGN. The behaviour of the cells under electrical stimulation needs also thorough investigation in future studies as well as possible migration of cells off the surface under magnetic field and without magnetic field. Finally, *in vivo* investigations with model electrodes are necessary to validate the practicality of this approach in the inner ear. In the present study however, most of the beads were found to be located in the cytosol rather than on the cell surface as demonstrated via AFM. We assume that beads were internalized via endocytosis already 24 and 48 h after the labelling of the cells was carried out. Thus, this presents a promising approach for the long-term drug delivery from the implant surface via cells attached by magnetic beads.
